# Immune checkpoint inhibitors in EGFR-mutation positive TKI-treated patients with advanced non-small-cell lung cancer network meta-analysis

**DOI:** 10.18632/oncotarget.26541

**Published:** 2019-01-04

**Authors:** Luigi Cavanna, Chiara Citterio, Elena Orlandi

**Affiliations:** ^1^ Oncology and Hematology Department, Oncology Unit, Piacenza General Hospital, Piacenza, Italy

**Keywords:** immuno-oncology, immunotherapy, NSCLC, EGFR mutation, chemotherapy

## Abstract

Non-Small Cell Lung Cancer (NSCLC) patients with Epidermal Growth Factor Receptor (EGFR) mutation benefit from a first line of treatment with tyrosine kinase inhibitors (TKIs). After progression, the choice of treatment is between chemotherapy and immune checkpoint inhibitors, but the role of EGFR mutation in the response to immunotherapy is still unclear. A network meta-analysis was performed and 4 randomized trials comparing immune checkpoint inhibitors versus chemotherapy were identified. A Bayesian network meta-analysis was carried out to compare three checkpoint inhibitors (nivolumab, pembrolizumab and atezolizumab) versus chemotherapy (docetaxel), evaluating their Hazard Ratio (HR) and 95% Confidence Interval (CI) for Overall Survival (OS). Results suggest that patients with NSCLC and EGFR mutation, previously treated with TKIs, show better OS when treated with docetaxel in comparison to checkpoint inhibitors treatment.

## INTRODUCTION

Activating Epidermal Growth Factor Receptor (EGFR) mutations in western countries are found in approximately 15% of patients with lung adenocarcinomas [1]. Erlotinib, gefitinib, and afatinib, small-molecule EGFR tyrosine kinase inhibitors (TKIs) are a highly effective treatment for this population and is approved by the United States Food and Drug Administration (FDA) and the European Medicines Agency (EMA) for first-line use in patients with EGFR-mutant metastatic lung cancer.

Unfortunately, after approximately 12 months of these treatments, the majority of these patients’ experience progression; a second EGFR mutation in exon 20, T790M, can be found in approximately half of the patients, where it is believed to be the primary mediator of such resistance. In these cases, third-generation TKIs (such as osimertinib) are the effective salvage therapy, but when T790M is not found, chemotherapy remains the standard option for these patients [2].

Checkpoint inhibitors-based combination therapies are emerging as a new therapeutic modality in non-small-cell lung cancer (NSCLC), but their role in EGFR-mutant adenocarcinomas is unclear. Immunotherapic agents such as nivolumab [3] and pembrolizumab [4], two programmed cell death-1 (PD-1) immune checkpoint inhibitors and atezolizumab [5], a humanized antiprogrammed death ligand-1 (PD-L1) monoclonal antibody which inhibits PD-L1 could be considered as potential alternative salvage treatments for patients previously treated with TKIs and without T790M.

The objectives of this review are to compare the results of second-line immunotherapy (nivolumab, pembrolizumab, atezolizumab) with the standard second-line chemotherapy (docetaxel) in term of OS (overall survival) Hazard Ratio (HR) in previously treated TKIs patients with EGFR mutation. To our knowledge this review is the first network meta-analysis performed about this topic in the English literature.

## RESULTS

**Figure 1:**
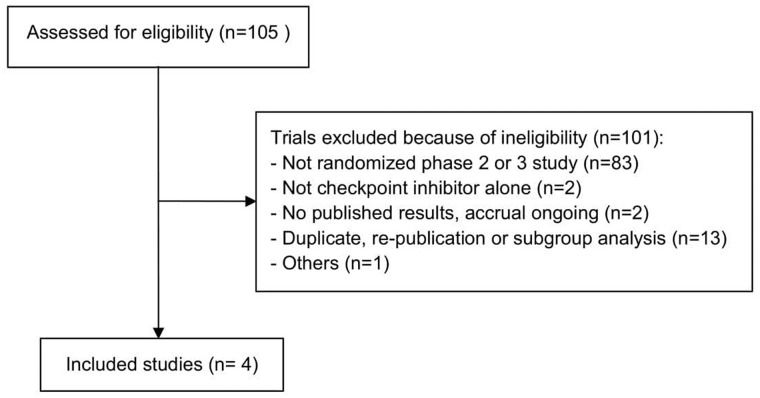
Flow chart illustrating the result of the online search and articles selection

After selecting abstracts and titles we identified 4 studies (Figure 1); they involved a total of 2,753 patients, 272 of then (9.88%) with known EGFR mutation, treated with checkpoint inhibitors (nivolumab (n=44 patients), pembrolizumab (n=60 patients) and atezolizumab (n=53 patients)) or docetaxel (n=115 patients) as second- or third-line therapies after disease recurrence or progression during or after TKI treatment.

The second-line chemotherapy drugs studied were: nivolumab, docetaxel, and atezolizumab. We had data about the direct comparisons between nivolumab and docetaxel (one study) and pembrolizumab and docetaxel (one study); data from two studies for the comparisons between atezolizumab and docetaxel. Eligible patients had: sensitive EGFR mutations, for a total of 272 patients (9.88% of the 2,753 included in the 4 studies), disease recurrence or progression during or after TKIs treatment.

OS, evaluated on the whole sample (EGFR mutated and not mutated) of 2,753 patients, is higher for patients treated with checkpoint inhibitors compared with patients treated with docetaxel, however in the subgroup of patients (n=272) with EGFR mutation, the OS of patients treated with docetaxel is higher (HR≥1) in comparison with nivolumab and atezolizumab (HR=1.18, 1.24 and 0.99) for patients treated with pembrolizumab we have an HR of 0.88 which could be explained by the higher number of patients in the pembrolizumab arm (n=60) compared to the docetaxel arm (n=26). The subgroup analyses for EGFR mutated is well represented in the forest plot of the article of Rittmeyer et al. [5].

**Figure 2:**
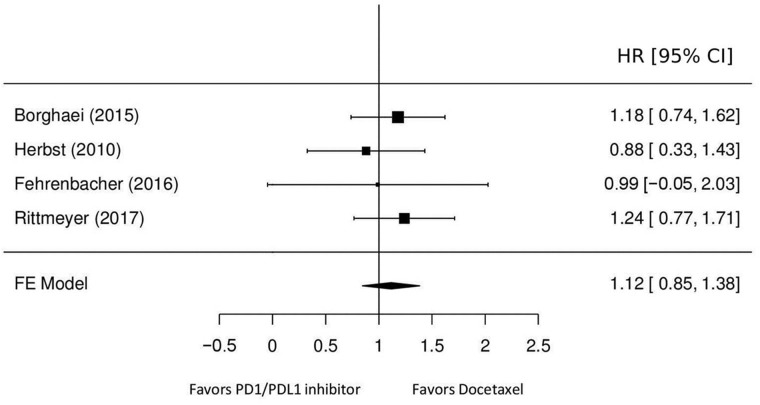
Hazard ratios of OS for patients treated with immune checkpoint inhibitors, compared with those treated with chemotherapy

**Figure 3:**
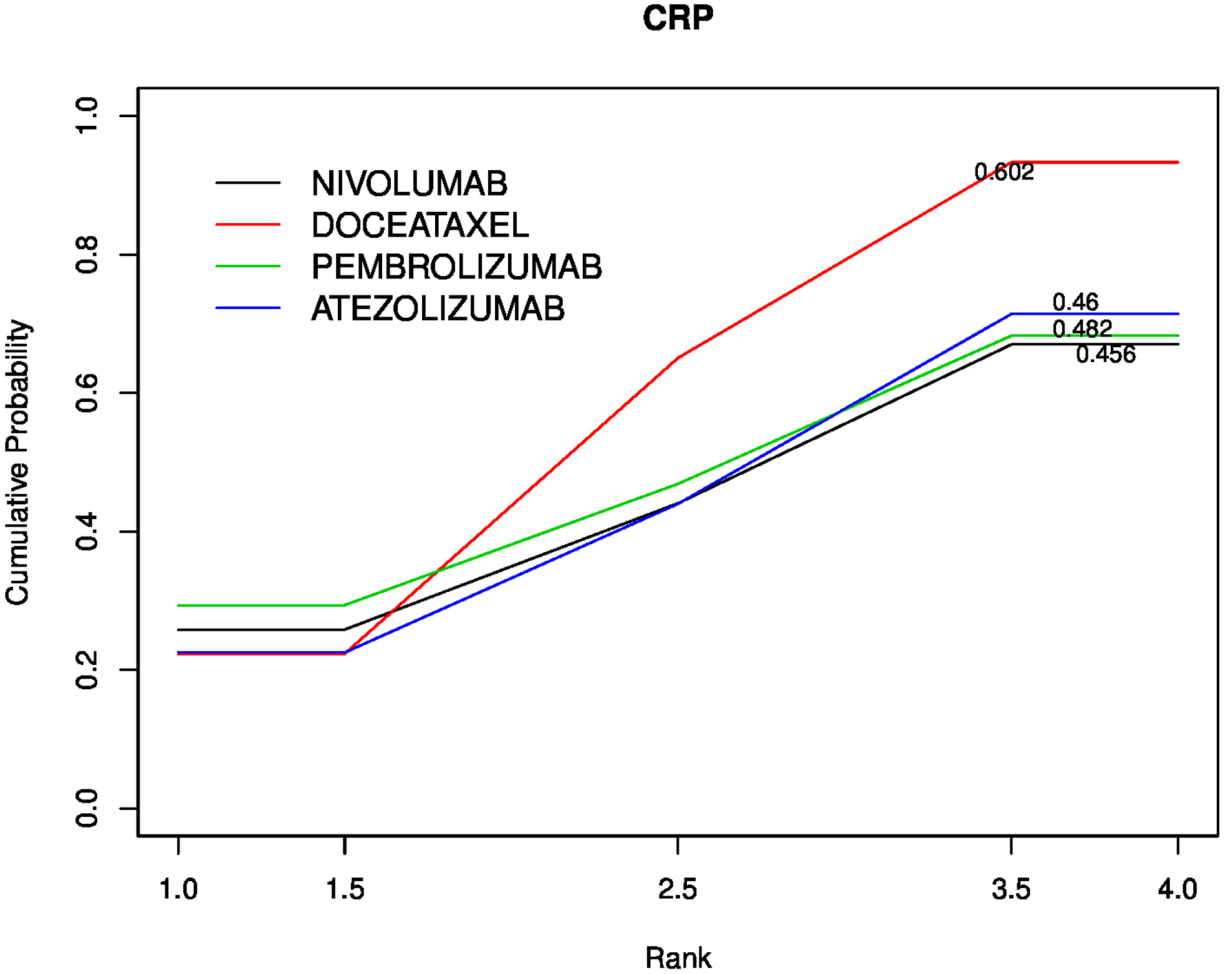
Cumulative probability of the treatment rank and SUCRA for the 4 treatments from the network meta-analysis on OS HR

The forest plot (Figure 2) evidence a pooled HR of 1.12 (95% CI: 0.85-1.38; heterogeneity p=0.94) and a statistically significant treatment effect (p<0.0001). Figure 3 reports the results of the network meta-analyses with SUCRA (SUrface under the Cumulative RAnking curve) values used to rank the four evaluated treatments [6]. The greater the value of the SUCRA, i.e. the greater the portion of area under the curve, the better the treatment performance. The median of the I^2^ distribution is 70% (0%-96%), I^2^>50% demonstrated significantly statistical heterogeneity. The results show that the most effective therapy is docetaxel (SUCRA=60%), the SUCRA for the remaining treatments are similar: pembrolizumab (SUCRA=48%), atezolizumab (SUCRA=46%) and nivolumab (SUCRA=45.6%).

## DISCUSSION

This meta-analysis has demonstrated that patients with NSCLC and EGFR mutation status have a better response in term of OS when treated with docetaxel compared with checkpoint inhibitor as second- or third-line therapy after disease recurrence or progression during TKI treatment.

The present network meta-analysis indicates the presence of a certain heterogeneity between studies (I^2^= 70%) but given the small number of studies available for each comparison it is difficult to make a hypothesis about the origin of this heterogeneity. It must be underlined that in this review patients’ quality of life and toxicities of the treatment schemes were not considered. Recent studies [7] evidence that immune checkpoint inhibitors significantly prolonged OS when compared with docetaxel chemotherapy overall in patient EGFR wild type but not in EGFR mutant patients and they demonstrated that EGFR mutation status is a potential predictive biomarker for OS in advanced NSCLC treated with an immune checkpoint inhibitor versus docetaxel. Lee et al. [8] concluded that in EGFR-mutant advanced NSCLC, immune checkpoint inhibitors do not improve OS over that with docetaxel. Although the positive trend in OS benefit in patients with EGFR wild-type versus EGFR-mutant tumors, there were no statistically significant differences in median OS between the subgroups. Biomarkers could be used to select patients more likely to benefit from immune checkpoint inhibitors therapy. It must be emphasized that mutation burden is considered predictive of a benefit for checkpoint inhibition and EGFR-mutated lung cancer was shown to have low mutation burden [9]; on the other hand, in the Italian cohort of the Nivolumab Expanded Access Program, the presence of EGFR mutations seems to affect short-term (Overall Response Rate (ORR) and Dynamic Condition Response (DCR)) but not medium- (Progression Free Survival (PFS)) or long-term (Overall Survival (OS)) outcomes [10]. In this study [10] patients’ smoking habits were also examined, and it is suggested that NSCLC subgroups (never-smokers EGFR wild type and smokers with EGFR mutant) could benefit from nivolumab treatment and the reason could be a different mutational burden or PD-L1 expression. Recently, the results of five years’ follow-up of nivolumab in previously treated advanced NSCLC have been reported [11]. A total of 16 patients between all treated patients (n=129) had an OS ≥ 5 years after starting nivolumab. 69 of these 129 patients (53.3%) were tested for EGFR-mutation and 13 (18.8%) were EGFR-mutation positive. Two of the 5 years survivors 16 patients (12.5%) had EGFR mutation including an exon 20 insertion mutation and an exon 18 missense mutation. One of these 2 patients received no prior EGFR TKI therapy and one received erlotinib for about 3 months before starting nivolumab.

The performed network meta-analysis (Figure 3) demonstrates that checkpoint inhibitors have similar performance in terms of SUCRA (Pembrolizumab 48%, atezolizumab 46% and nivolumab 45.6%) and that docetaxel is the most effective therapy (SUCRA=60%).

The main limitation of this meta-analysis is the small number of studies which report results from clinical trial comparing second- and third-line therapies for NSCLC with available EGFR mutations status. Different mutations of the EGFR gene in different tumors have different immunogenicity, causing different responses to immune checkpoint therapy. Recent studies of NSCLC patients treated with pembrolizumab showed a different mutation burden [10, 12] and that immunogenicity of EGFR-mutant tumors could be increased with combination treatments [13] but more trials are required to confirm this finding. Other limitations are the number of prior TKI therapies between patients randomized to receive docetaxel or checkpoint inhibitors and the lack of data about smoking habits. To confirm the different response to docetaxel or checkpoint inhibitors, further randomized studies in patients with NSCLC previously treated with TKIs which report the type of EGFR mutation, universally determined by means of centralized testing or precise operative procedures, are needed. In conclusion, this network meta-analysis, with the limitations reported above seems to confirm the lesser efficacy of immunotherapy for NSCLC with EGFR-mutated patients previously treated with TKIs compared with chemotherapy.

## MATERIALS AND METHODS

### Study identification and data extraction

The search was based on the following keywords: NSCLC, EGFR, and immunotherapy. MEDLINE, PubMed, http://clinicaltrials.gov and American Society of Clinical Oncology (ASCO) were searched for published randomized controlled trials. Only randomized trials were analyzed; studies were phase II and III randomized trials of different second- and third-line checkpoint inhibitors for NSCLC previously treated with TKIs, with available EGFR mutations; a total of 4 studies were considered eligible for further evaluations (Table 1). The data extracted from the trials were: authors and year of publication, patient number, treatment comparison, number of patients for the checkpoint inhibitor arm and for the docetaxel arm, median OS, percentage of EGFR-mutated patients, OS, HR and 95% Confidence Intervals (95% CI). Data extraction was conducted by 2 reviewers.

**Table 1:** Characteristics of the studies included in the network meta-analysis

Author (year)	Total patients n.	EGFR Mutation (%)	Treatment comparisions	Median Overall Survival (mo)	Immunotherapy patients n. (%)	Docetaxel patients n. (%)	OS HR [95% CI]
Borghaei et al. (2015)	582	82 (14.1)	Nivolumab vs docetaxel	12.2 vs. 9.4	44 (56.66)	38 (46.34)	1.18 [0.69-2.00]
Herbst et al. (2016)	1034	86 (8.3)	Pembrolizumab vs docetaxel	10.4^A^ vs. 12.7^B^ vs. 8.5	60 (69.77)	26 (30.23)	0.88 [0.45-1.70]
Fehrenbacher et al. (2016)	287	19 (6.6)	Atezolizumab vs docetaxel	12.6 vs. 9.7	11 (57.90)	8 (42.10)	0.99 [0.29-3.40]
Rittmeyer et al. (2017)	850	85 (10)	Atezolizumab vs docetaxel	13.8 vs 9.6	42 (49.41)	43 (50.59)	1.24 [0.71-2.18]

^A^Pembrolizumab, 2 mg/kg, arm.

^B^Pembrolizumab, 10 mg/kg, arm.

### Statistical analysis

**Figure 4:**
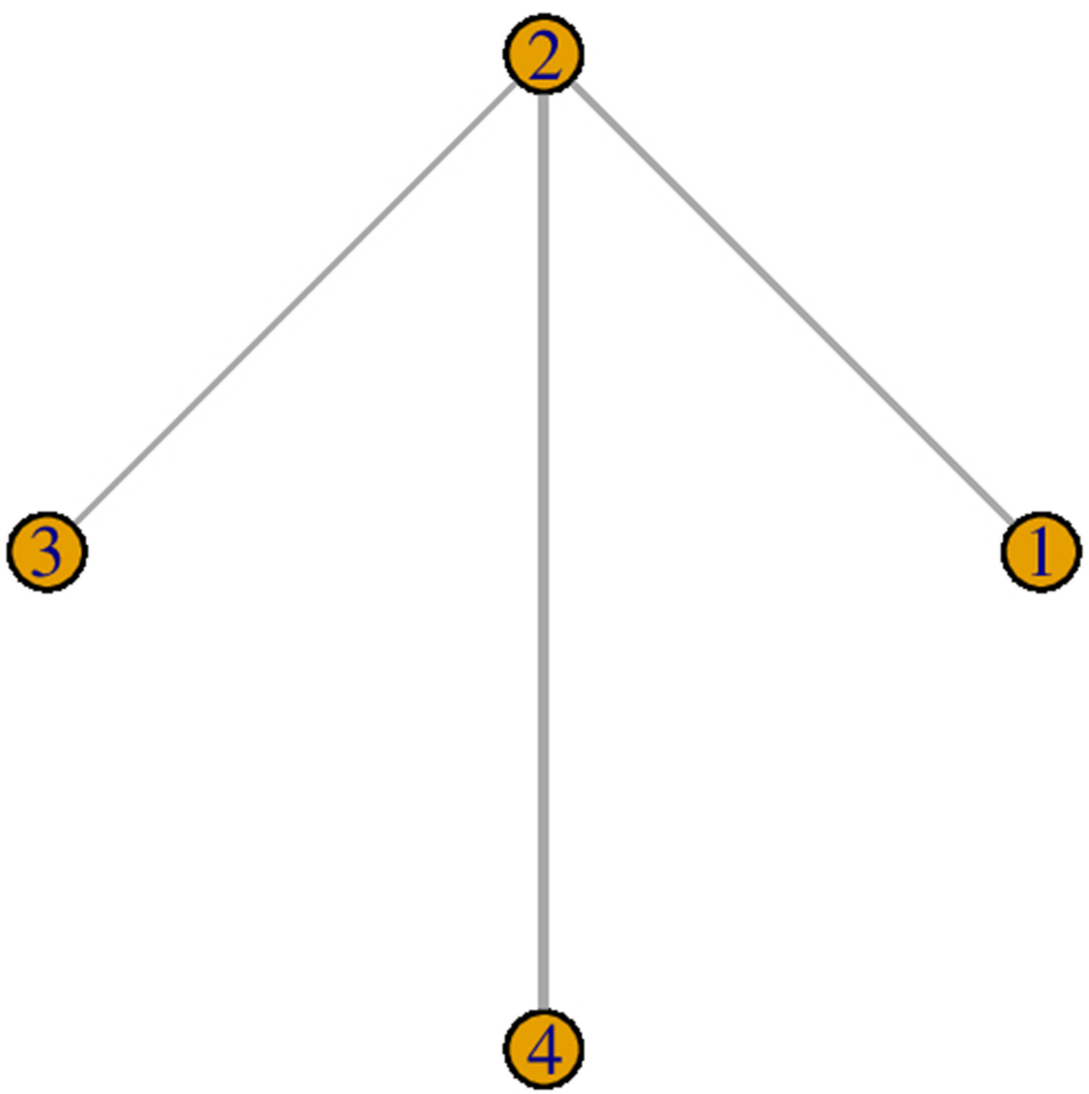
Study network

We focused on the OS. The analyzed outcome was OS HR with corresponding 95% CI. We did not observe comparisons which could be combined to make indirect inferences on a third observed comparison (no closed loops in Figure 4) [10, 14–16], so model used assumed consistency between direct and indirect evidence. Meta-analysis with a fixed-effect model was performed for the comparison between checkpoint inhibitors and docetaxel (Figure 4).

We ranked multiple treatments with a network meta-analysis using a Bayesian approach of easier computation and programming and to have a more reliable estimates of the heterogeneity present in the network. I^2^ statistic [17] is the method used to estimate heterogeneity. We transform the average rank in the cumulative probability of the treatment rank calculating the SUCRA (the area under the curve), the measure of the performance of treatment compared to the others. The greater the area under the curve (SUCRA) the better the effectiveness of the treatment [18]. Network meta-analysis was performed using R software (Core Team 2017) and the R2WinBUGS package.

## Abbreviations

ASCO: American Society of Clinical Oncology
CI: Confidence Interval
EGFR: Epidermal Growth Factor Receptor
EMA: European Medicines Agency
FDA: Food and Drug Administration
HR: Hazard Ratio
NSCLC: Non-Small Cell Lung Cancer
OS: Overall Survival
SUCRA: SUrface under the Cumulative RAnking curve
TKIs: Tyrosine kinase inhibitors
